# Production and Bioseparation Applications of Polyhydroxyalkanoate Nano-Granules Functionalized with Streptavidin

**DOI:** 10.3390/microorganisms13020312

**Published:** 2025-02-01

**Authors:** Yuyan Zhang, Jiping Zhao, Hui Guo, Xiaoyun Lu, Dan Tan

**Affiliations:** 1 School of Life Science & Technology, Xinjiang University, Urumchi 830049, China; zhangyy@xju.edu.cn; 2 Key Laboratory of Biomedical Information Engineering of the Ministry of Education, Department of Biological Science and Bioengineering, School of Life Science and Technology, Xi’an Jiaotong University, Xi’an 710049, China; zjp93823@sina.com (J.Z.); ljl122366@163.com (H.G.)

**Keywords:** polyhydroxyalkanoate nano-granules, bioseparation, streptavidin, biotin, mRNA isolation, magnetic recovery

## Abstract

Rapidly growing industrial biotechnology and bio-manufacturing require simple and cost-effective bioseparation tools. A novel strategy of bioseparation based on the streptavidin-decorated polyhydroxyalkanoate (PHA) nano-granules was developed in this study. By fusing to the N-terminus of PHA-associated phasin protein, the streptavidin was one-step immobilized on the surface of PHA nano-granules simultaneously with the accumulation of PHA in recombinant *Escherichia coli*. About 1.95 g/L of PHA nano-granules (54.51 wt% of cell dry weight) were produced after 48 h bacterial cultivation. The following qualitative and quantitative characterizations demonstrated that the streptavidin accounted for approximately 6.78% of the total weight of the purified PHA nano-granules and confirmed a considerable biotin affinity of 0.1 ng biotin/μg surface protein. As a proof of concept, the nano-granules were further functionalized with biotinylated oligo(dT) for mRNA isolation and about 1.26 μg of mRNA (occupied 2.59%) was purified from 48.45 μg of total RNA, achieving good integrity and high purity with few DNA and rRNA contaminations. Moreover, the nano-granules retained more than 80% of their initial mRNA recovery efficiency after ten cycles of repeated use. The PHA-SAP nano-granules were also functionalized with biotinylated magnetic beads, allowing magnetic recovery of the PHA nano-granules from cell lysates that still needs optimization. Our study provides a novel and expandable platform of PHA nano-granules that can be further functionalized with various biological groups for bioseparation applications. The functional PHA nano-granules have a great potential to serve as bioseparation resin for large-scale purification processes after suitable optimizations for “bench-to-factory” translation, contributing to scalable and sustainable bioprocessing.

## 1. Introduction

Bioseparation is a series of downstream processes to purify proteins, nucleic acids, and other molecules from biological crude samples after fermentation, which includes the centrifugation for isolation of biomass and extracellular products, cell disruption for intracellular products, and further purification via extraction, electrophoresis, chromatography, crystallization, etc. [1]. The downstream bioseparation steps shift the overall manufacturing costs, and efforts have been made to develop simple, consistent, and cost-effective purification strategies [2]. Favorably, magnetic separation is one of the most versatile separation processes in biotechnology because of its fast and gentle process and its easy scale-up and automation [3,4].

Streptavidin is usually a tetrameric protein with symmetrically arranged subunits and exhibits a remarkably high affinity with biotin, which is one of nature’s strongest non-covalent bindings [5]. Because of its specificity, sensitivity, and stability, the streptavidin–biotin system has been widely used in a variety of non-invasive applications for bioseparation, labeling, and qualitative and quantitative detection of biomolecules [6]. Among them, the coupled interaction of antigen–antibody with streptavidin–biotin has been extensively studied and applied in affinity chromatography and diagnosis. Chemically labeling the proteins with streptavidin or biotin is also commonly employed to study the expression and cellular localization of various biomolecules on the cell surface or inside the cell [7]. However, the preparation of streptavidin immobilized chromatography or beads for bioseparation purposes requires tedious procedures, including the production and purification of streptavidin, and the chemical cross-linking step with synthetic polymer beads or cellulose, which will often lead to the loss of streptavidin activity [8].

A distinct one-step immobilization of various proteins on the surface of polyhydroxyalkanoate (PHA) nano-granules has provided an alternative for the preparation of streptavidin-decorated beads [9,10]. PHAs are a family of biodegradable and biocompatible polyesters synthesized by various microorganisms in the form of insoluble intracellular nano-granules with diameters of 50–500 nm [11]. PHAs are surrounded by several granule-associated proteins, including PHA synthase (PhaC), phasin (PhaP), and depolymerase (PhaZ) etc., forming a “core-shell” structure [12]. By fusion to the N-terminus of PhaC or PhaP, proteins of interest can be attached to the surface of PHA with the assembly of nano-beads [13,14]. These polymer inclusions can be isolated and successfully maintained as nano-granules outside the cell while exhibiting the protein function at a high density, which has been investigated as a novel strategy for oriented enzyme immobilization. Functional PHA nano-granules have been applied in imaging, protein purification, bioseparation, early sensitive diagnostics, and even pandemic vaccines [9,15,16]. Recently, PHA beads with a fusion of PhaC and streptavidin were reported to be capable of binding biotin with a remarkable binding capacity, but the yield of PHA beads was relatively low, mainly due to the interference of the catalytic function of PhaC and the formation of PHA [8]. Thus, the PHA phasin protein PhaP, which is not responsible for PHA synthesis and has strong interactions with PHA granules, is more preferred for the immobilization of the target protein.

**Figure 1:**
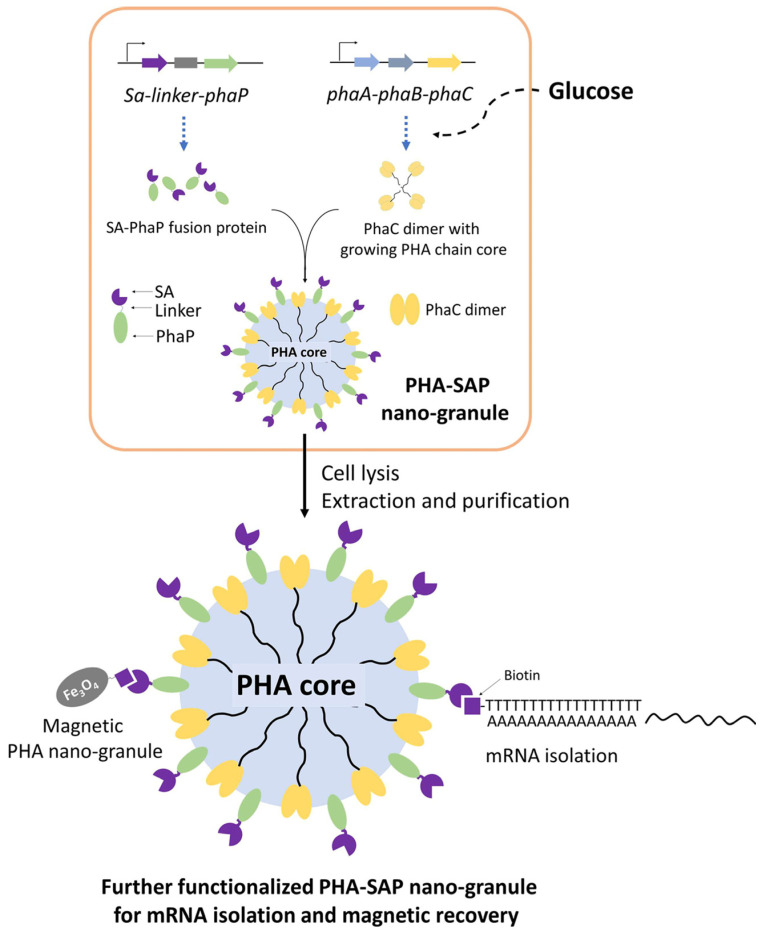
One-step preparation of streptavidin-decorated PHA nano-granules with their applications in mRNA isolation and magnetic recovery. SA: Streptavidin, PhaP: PHA phasin protein, PhaC: PHA synthetase, PhaA: NADPH-dependent acetyl-CoA acetyltransferase, PhaB: 3-Hydroxybutyryl-CoA dehydrogenase.

In this study, streptavidin-decorated PHA nano-granules were designed and produced in recombinant *E. coli* by fusing streptavidin to the N-terminus of PhaP, which were purified and qualitatively and quantitatively characterized for their affinity to biotin. In addition, streptavidin-decorated nano-granules were further functionalized with oligo(dT) for mRNA isolation or prepared as magnetic granules for their fast recovery. Our study provides novel functional PHA nano-granules for possible and various applications in bioseparation (Figure 1).

## 2. Material and Methods

### 2.1. Microorganisms, Plasmids, and Culture Conditions

All strains and plasmids used in this study are listed in Table 1. *E. coli* DH5α (Takara, Kyoto, Japan) was used for plasmid construction and cloning. *E. coli* BL21(DE3) (Novagen, Madison, WI, USA) was used as the host for protein expression and PHA production. The plasmid pET28a (Invitrogen, Carlsbad, CA, USA) was used for efficient expression of proteins in *E. coli*, and pCDF-ABC was a previously constructed plasmid for the production of PHA nano-granules [17]. All the *E. coli* strains were cultured in LB medium at 200 rpm and 37 °C and were supplemented with kanamycin (50 μg/mL) or streptomycin (100 μg/mL) when necessary.

### 2.2. Plasmid and Strain Constructions for the Production of PHA Nano-Granules Decorated with Streptavidin (Shorted as PHA-SAP Nano-Granules)

The plasmid pET28a-SAP was constructed for the expression of the streptavidin-PhaP fusion protein. The monomeric streptavidin gene [18] from *Streptomyces Avidinii* with a linker sequence (protein sequence: GGGGSGGGSGGS) in its 3′ end was synthesized by the BGI Company (Shenzhen, China), and 5′ fused to the *phaP* gene from *Aeromonas hydrophila* by overlap extension PCR. The *sa*-*linker*-*phaP* fragment was cloned into the vector pET28 between the *Nco* I and *Xho* I restriction sites under the control of the T7 promoter. Plasmid pCDF-ABC was constructed previously for the production of PHA nano-granules, which contained PHA synthetic gene cluster *pha*CAB encoding β-ketothiolysis (PhaA), acetoacetyl-CoA reductase (PhaB), and PHA synthase (PhaC) from *Ralstonia eutropha* under the T7 promoter [17]. All the genetic manipulations in this study are based on standard protocols or manufacturer’s instructions. Primers (Table 1) were synthesized by GENEWIZ Company (Nanjing, China).

Plasmids pET28a-SAP and pCDF-ABC were co-transformed into *E. coli* BL21(DE3) and confirmed by colony PCR. The recombinant *E. coli* was further cultivated and induced by 1 mM isopropyl β-d-1-thiogalactopyranoside (IPTG) and subjected to SDS-PAGE analysis to detect the expression of the streptavidin-PhaP fusion protein.

### 2.3. Production and Purification of PHA-SAP Nano-Granules

The recombinant *E. coli* BL21(DE3) harboring pET28a-SAP and pCDF-ABC was cultivated in a modified MMG medium (Table 2) for the synthesis of PHA nano-granules as previously reported [19]. A total of 1 mM of IPTG was added after 3 h of cultivation to induce the expression of the streptavidin-PhaP fusion protein. After 48 h of cultivation, cells were harvested for the determination of PHA content and the purification of PHA-SAP granules.

PHA granules were purified by mechanical cell disruption and glycerol-gradient-based ultracentrifugation [20]. Generally, cells were disrupted using a high-pressure homogenizer (PHD Technology LLC, Saint Paul, MN, USA) at 15,000 kpsi followed by ultracentrifugation (Eppendorf Himac CNX100, Hamburg, Germany) at 100,000× *g* and 10 °C for 2.5 h on a gradient containing 88% (*v*/*v*, bottom layer)-44% (*v*/*v*, upper layer) glycerol. After the white layer of PHA nano-granules above the 88% glycerol layer was collected and washed with 50 mM PBS buffer three times, the PHA granules were resuspended and lyophilized to powder.

For the following characterization of PHA-SAP nano-granules, purified PHA powder was dissolved in 50 mM PBS buffer (pH 7.5) and subjected to ultrasonication (Bilon, Tianjin, China) at 200 W, working 5 s with 15 s pause for a total of 5 min before use.

### 2.4. Characterization of PHA-SAP Nano-Granules

#### 2.4.1. Microscopic Observation of PHA Nano-Granules

The PHA-SAP nano-granules in the recombinant *E. coli* cells were visualized by fluorescence microscopy at an excitation wavelength of 460 nm after Nile blue A staining [21]. The morphology, distribution, and accumulation of intracellular PHA nano-granules can be directly observed by transmission electron microscopy (TEM; HITACHI, H-7650B, Tokyo, Japan).

#### 2.4.2. Determination of PHA Content by Gas Chromatography

Recombinant cells were harvested, washed, and lyophilized to measure the cell dry weight (CDW). About 40 mg lyophilized cells were subjected to esterification and then gas chromatography (GC) on a GC-500 system (PerkinElmer, Waltham, MA, USA) to determine the intracellular PHA content [22].

### 2.5. Identification of Streptavidin-PhaP Fusion Protein on the Surface of PHA-SAP Nano-Granules

#### 2.5.1. SDS-PAGE Analysis of Purified PHA-SAP Nano-Granules

A concentration of 10 mg/mL of PHA nano-granules was prepared and well suspended by ultrasonication and then analyzed using SDS-PAGE as reported [23].

#### 2.5.2. The Concentration of Total Proteins on the Surface of PHA Beads Was Determined by Bradford Analysis

The concentration of total protein on the surface of PHA nano-granules was determined using the Bradford method. Briefly, 10 mg/mL of PHA-SAP nano-granules was prepared, ultrasonically dispersed, and treated with Bradford agent. The absorbance at 595 nm was detected using Bovine serum albumin (BSA) as the standard [24].

#### 2.5.3. Qualitative Characterization of Biotin Binding Ability of PHA-SAP Nano-Granules via Western Blot

The Western blot between biotinylated horse radish peroxidase (biotin-HRP, ZeYe Bio, Shanghai, China) and the PHA-SAP nano-granules was conducted as previously reported [25]. Briefly, 50 mg/mL of PHA nano-granules was prepared and subjected to SDS-PAGE. The proteins on the surface of PHA-SAP nano-granules were transferred to a PVDF membrane (Millipore, Bedford, MA, USA) and then blocked with 5% BSA for 1 h. The transfer buffer TBST contained 250 mL of 10 mM PBS with 2 mL of tween-20 (pH 7.0). The streptavidin on the PHA surface was probed with 2.5 mg/mL of a biotin-HRP solution to ensure that the ratio of biotin-HRP and the total protein on the PHA-SAP surface reached over 10:1, as reported [8]. After incubation at room temperature for 1 h, the PVDF membrane was washed three times with TBST buffer (15 min for each round) and treated with the Clarity™ Western ECL Substrate (BIO-RAD, Hercules, CA, USA). Proteins were then detected using the FlorChem FC2 System (Alpha Innotech, San Antonio, TX, USA). An equal amount of blank PHA nano-granules without streptavidin-PhaP protein served as a negative control.

#### 2.5.4. Quantitative Characterization of Biotin Binding Ability of PHA-SAP Nano-Granules

Biotin-HRP was also used for the quantitative characterization of the biotin-binding capacity of PHA-SAP nano-granules via chromogenic reaction with tetramethylbenzidine (TMB) [26]. Different amounts of PHA-SAP nano-granules, 8.0 mg, 10.0 mg, 15.0 mg, 20.0 mg, and 40.0 mg, respectively, were added to the solution containing a fixed amount of biotin-HRP (1 μg), and incubated at room temperature for 1 h, followed by washing with PBS buffer for three times. A total of 400 μL of TMB, the substrate for HRP, was added for the chromogenic reaction at 37 °C for 30 min. The reaction was terminated by 2 mM H_2_SO_4_, followed by the detection of the absorbance at 450 nm. For the repeated use of PHA nano-granules, after one cycle of TMB chromogenic reaction, the yielded PHA-SAP-biotin-HRP nano-granules were recycled by centrifugation at 12,000 rpm and 4 °C for 2 min and subjected to the same reaction mixture and repeated for ten rounds. The initial value (OD_450nm_) was set to 100%.

### 2.6. Functionalization of PHA-SAP Nano-Granules with Oligo(dT) for mRNA Isolation

#### 2.6.1. Modification of PHA-SAP Nano-Granules with Oligo(dT)

PHA-SAP nano-granules were washed twice with 2 × SSC buffer (17.53 g/L of NaCl and 8.82 g/L of sodium citrate, pH 7.0) and homogeneously resuspended in 0.2 mL of 2 × SSC buffer to the final concentration of 10 mg/mL. An excessive 100 μM of biotinylated oligo(dT)_18_ probe (GENEWIZ, Nanjing, China) was added, and the mixture was incubated at room temperature for 1 h. After centrifugation at 12,000 rpm and 4 °C for 2 min, the mixture was washed 3 times and resuspended with 0.3 mL of 20 × SSC to generate a PHA-SAP-oligo(dT) nano-granules solution.

#### 2.6.2. mRNA Isolation by PHA-SAP-oligo(dT) Nano-Granules

The total RNA of rat C6 glioma cells was purified using an RNAprep PureCell Kit and was divided into three experimental samples for the parallel isolation procedure of mRNA. The mixture of total RNA and PHA-SAP-oligo(dT) nano-granules at a 1:1 ratio was heated at 65 °C for 10 min and then gradually cooled down to room temperature, annealing for another 10 min. After centrifugation, the supernatant was collected (supernatant 0), and the precipitation was washed 3 times with 2 × SSC buffer. The supernatants in the first and third washing steps were also collected for further tests (supernatants 1 and 3). The precipitated PHA granules with mRNA on the surface were washed twice with DEPC water to elute mRNA, followed by the purification of mRNA by ethanol precipitation. The concentration and purity of mRNA were determined using a Nanodrop Spectrophotometer (Thermofisher, Waltham, MA, USA) and further visualized by electrophoresis, in which all steps were prepared with DEPC water. To test the reusability of PHA-SAP-oligo(dT) nano-granules for mRNA isolation, after one cycle of mRNA recovery, the PHA-SAP nano-granules were recycled by centrifugation at 12,000 rpm and 4 °C for 2 min and subjected to the same purification procedure and repeated for ten rounds; the amount of purified mRNA in each cycle was detected. The initial amount of purified mRNA was set to 100%. All the results indicate the average value of three parallel experiments.

#### 2.6.3. Real-Time Quantitative PCR

Using the purified mRNA as the template, the real-time qPCR was conducted to test the specifically expressed gene S100B in C6 cells as well as the IL-10 gene, which is not expressed in C6 cells (negative control) [27]. The cDNA was synthesized using Evo M-MLV RT Premix. A real-time qPCR assay was performed using a ChamQ SYBR qPCR Master Mix (SYBR Green I), using the GADPH gene as the inner standard. All manipulations were conducted based on the previously described protocol [28].

### 2.7. Preparation of the Magnetic PHA Nano-Granules for the Fast Recovery

The biotin-labeled Fe_3_O_4_ magnetic beads were prepared by Shanghai Nano Research Institute (Shanghai, China) with a size of 14 ± 6 nm. The recombinant *E. coli* (pCDF-ABC) (pET28a-SAP) were cultured for 48 h for the production of PHA-SAP nano-granules, followed by cell lysis using high-pressure homogenization. A total of 5 mg of the ultrasonically homogenized biotin-Fe_3_O_4_ was added to 50 mL of the above cell lysate, which was incubated at 37 °C for 1 h. A magnetic separator was applied to recover the magnetic PHA nano-granules and washed 3 times using PBS buffer. The yielding magnetic PHA granules were lyophilized and used for GC analysis, Nile Blue staining, and TEM observation. The supernatants, including the ones in the wash procedure, were also collected for further GC detection. The same 50 mL cell lysate was directly lyophilized as a control to determine its total amount of PHA. All measurements were conducted in triplicate.

## 3. Results

### 3.1. Construction and Verification of the Producer of PHA-SAP Nano-Granules

**Figure 2:**
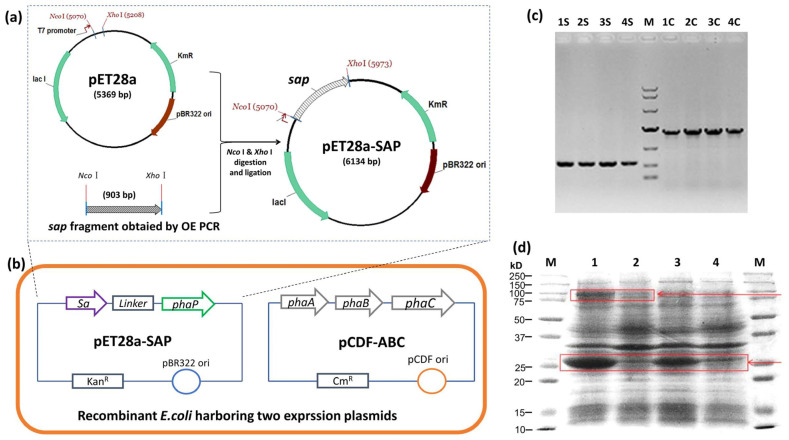
Strain and plasmid construction for the production of PHA-SAP nano-granules. (**a**) Construction of pET28a-SAP plasmid for the expression of streptavidin-PhaP fusion protein. (**b**) Construction of recombinant *E. coli* strain harboring plasmids pET28a-SAP and pCDF-ABC that contained PHA synthetic gene cluster *pha*CAB. (**c**) Colony PCR verification of recombinant *E. coli* harboring two expression vectors. Lane 1S–4S: colony PCR results of *sap* fragment of four different clones, Lane 1C–4C: colony PCR results of *phaC* gene of the same four clones with 1S–4S. M: DNA ladder, top to bottom, was 10,000, 7000, 4000, 2000, 1000, 500, 250 (bp). (**d**) Protein profile of recombinant *E. coli* harboring two expression vectors after IPTG induction. Lane 1 and Lane 2: Protein profile of recombinant *E. coli* harboring pET28a-SAP and pCDF-ABC with (Lane 1) and without (Lane 2) IPTG induction. Lane 3 and Lane 4: Protein profile of recombinant *E. coli* harboring pET28a-SAP with (Lane 3) and without (Lane 4) IPTG induction. Arrows indicate the streptavidin-PhaP fusion protein (lower one) and the PhaC protein (upper one).

The streptavidin gene *sa* was synthesized with a linker sequence, 5′ fused to the *phaP* gene by OE PCR, and ligated to pET28a to yield pET28a-SAP (Figure 2a). The pET28a-SAP and PHA synthetic plasmid pCDF-ABC were co-transformed into *E. coli* BL21(DE3), and the colony PCR result showed the clear *sa-phaP* (~900 bp) and *phaC* (~1.9 kb) bands, indicating that two plasmids were successfully transferred into *E. coli* (Figure 2b,c). The protein profile of the recombinant *E. coli* was also detected after IPTG induction, which showed the efficient expression of streptavidin-PhaP fusion protein and PHA synthase PhaC (Figure 2d), confirming the functionality of the recombinant strain harboring two plasmids for the production of PHA nano-granules.

### 3.2. Production and Characterization of PHA-SAP Nano-Granules

PHA nano-granules have been developed as a novel strategy for in situ immobilization of enzymes [14]. The recombinant *E. coli* harboring pET28a-SAP and pCDF-ABC was expected to produce PHA nano-granules with streptavidin immobilization on the surface in situ and in one step. The heterologous expressed PhaA, PhaB, and PhaC from *R. eutropha* in pCDF-ABC are essential for the formation of PHA nano-granules using the precursor glucose. The streptavidin was N-terminally fused to PhaP from *R. eutropha* by a designed flexible linker so that the streptavidin-PhaP fusion protein can be anchored onto the surface of PHA nano-granules simultaneously with the accumulation of PHA via the interaction of the amphipathic PhaP with PHA granules [13] (Figure 1).

**Figure 3:**
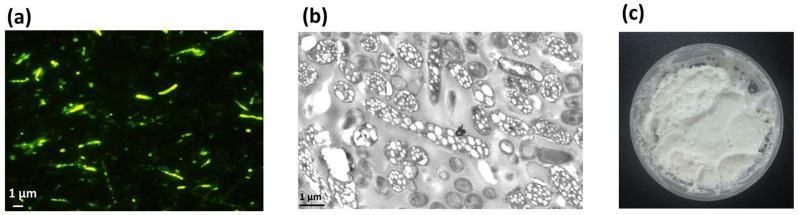
Production and characterization of streptavidin decorated PHA-SAP nano-granules. (**a**) Fluorescence microscopy of PHA-producing *E. coli* harboring two expression plasmids at an excitation wavelength of 460 nm after Nile blue A staining. (**b**) TEM observation of PHA-containing *E. coli* harboring two expression plasmids, showing conspicuous white PHA nano-granules in the cells. (**c**) Purified white powder of PHA-SAP nano-granules.

The recombinant *E. coli* cells were cultured in modified MMG medium for 48 h, and the cell dry weight (CDW) was 3.58 g/L with a PHA content of 54.51 wt%, resulting in a yield of 1.95 g/L, as determined by GC analysis. The formation of PHA nano-granules in cells was further confirmed by fluorescence microscopy and transmission electron microscopy (TEM). Cells containing PHA granules exhibited bright orange fluorescence at an excitation wavelength of 460 nm after Nile Blue A staining (Figure 3a). TEM observation also clearly demonstrated the presence of numerous white PHA nano-granules in the recombinant cells (Figure 3b). The recombinant cells were harvested and mechanically disrupted, followed by the purification of PHA by glycerol gradient-based ultracentrifugation. The purified PHA nano-granules were lyophilized to be the white powder, with a purity reaching more than 90% as determined by GC analysis (Figure 3c). All the above results demonstrated the remarkable accumulation of PHA nano-granules in recombinant cells without affecting cell growth.

### 3.3. Qualitative and Quantitative Characterization of Streptavidin-PhaP Fusion Protein on the Surface of PHA-SAP Nano-Granules

**Figure 4:**
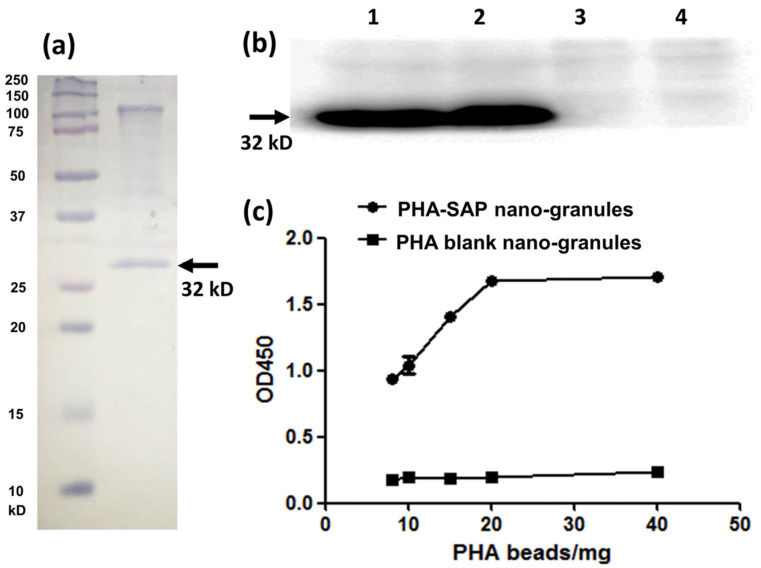
Qualitative and quantitative characterization of the streptavidin-PhaP fusion protein on the surface of PHA-SAP nano-granules. (**a**) SDS-PAGE analysis of the protein profile on the surface of the PHA-SAP nano-granules, with streptavidin-PhaP fusion protein being the dominant one (arrow). (**b**) Qualitative characterization of biotin-binding ability of PHA-SAP nano-granules via Western blot. Lane 1 and Lane 2: two repeated parallel tests of biotin affinity of PHA-SAP nano-granules. Lane 3 and Lane 4: two repeated parallel tests of biotin affinity of PHA blank granules without streptavidin on the surface. (**c**) Quantitative characterization of biotin affinity with PHA-SAP nano-granules after chromogenic reaction with TMB.

The purified PHA-SAP powder was subjected to SDS-PAGE analysis, and the protein profile on the surface of PHA granules indicated a dominant band of streptavidin-PhaP fusion protein (~32 kD), with an upper band of native PhaC that is covalently connected to the PHA nano-granules [29] (Figure 4a). The concentration of total protein on the surface of PHA beads was further determined by Bradford analysis. Using BSA as a standard, a standard curve was obtained as Y = 1.2184X + 0.3631, where Y is the OD_595nm,_ and X is the protein concentration. Thus, the concentration of the total surface protein (streptavidin-PhaP fusion protein and other proteins on the surface) was calculated to be 2.74 μg/mg PHA granules. In addition, the hydrophobic PHA nano-granule itself was found not to affect the detection of the surface protein profile and the total concentration by Bradford assay [21].

The surface-displayed streptavidin protein on PHA nano-granules was further qualitatively and quantitatively confirmed by its affinity with biotin-HRP. Western blot was used for qualitative identification of their biotin-binding ability (Figure 4b). After the surface proteins on the PHA granules were transferred to the PVDF membrane, an excessive biotin-HRP solution (the ratio of biotin-HRP and total proteins on the PHA surface was over 10:1) was added to allow the interaction of streptavidin and biotin. As is shown in Figure 4b, a significant band at about 32 kD was observed in the PHA-SAP nano-granules after chemiluminescence (Lane 1, 2), while no bands in the PHA blank granules (Lane 3, 4), demonstrating the functionality of the surface-displayed streptavidin on PHA-SAP nano-granules.

The HRP-biotin binding ability of PHA-SAP beads was further quantitatively characterized by the absorbance at 450 nm after chromogenic reaction with TMB [26]. Compared to the PHA blank granules, which showed little binding with biotin-HRP, the affinity of PHA-SAP granules and biotin-HRP gradually increased with the amount of PHA granules (Figure 4c). It reached the highest level and was stable when 20 mg or more of PHA-SAP granules were added, indicating that the binding of streptavidin and biotin reached saturation at 20 mg of PHA beads (equivalent to 54.80 μg of total surface protein) when 1 μg of biotin-HRP was used. Therefore, the average binding ability of streptavidin-decorated PHA-SAP nano-granules was 50 ng of biotin-HRP/mg PHA beads, which was equivalent to 0.1 ng biotin/μg surface protein. Thus, the amount of streptavidin protein on the surface of PHA granules was calculated to be 67.81 μg/mg PHA, indicating the streptavidin accounted for approximately 6.78% of the total weight of the purified PHA nano-granules.

All the above results demonstrated that the streptavidin was successfully anchored to the surface of PHA nano-granules via streptavidin-PhaP fusion protein without affecting the accumulation of PHA, and the yielded PHA-SAP nano-granules exhibited considerable surface density of streptavidin and a high affinity with biotin, which is the basis of further functionalization.

### 3.4. Functionalization of PHA-SAP Nano-Granules with Oligo(dT) for mRNA Isolation

The separation of mRNA is commonly based on the interaction of its poly (A) tail and immobilized oligo(dT) in affinity chromatography or beads [30]. In our study, the oligo(dT) was anchored on the surface of PHA nano-granules via the interaction between streptavidin on the PHA surface and the biotinylated oligo(dT), yielding PHA-SAP-oligo(dT) granules for the isolation and purification of mRNA from C6 cells.

**Figure 5:**
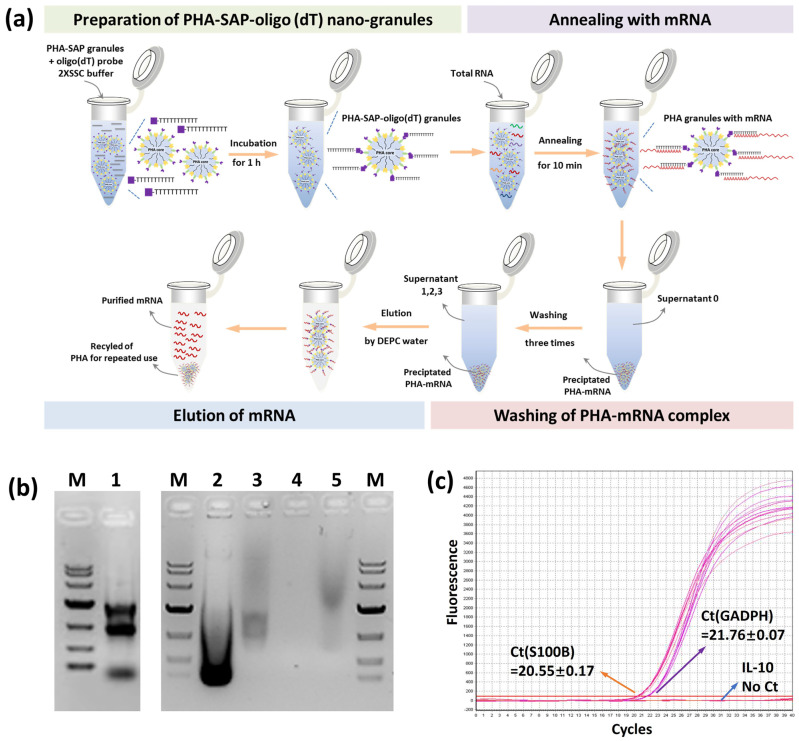
mRNA isolation by PHA-SAP nano-granules further functionalized with oligo(dT). (**a**) The scheme of mRNA isolation procedure by PHA-SAP nano-granules. (**b**) The electrophoresis detection of purified mRNA and other intermediates. Lane 1: Total RNA of C6 cells. Lane 2: The supernatant after precipitation of mRNA with PHA-SAP-oligo(dT) granules (supernatant 0). Lane 3: The supernatant after first washing of precipitated mRNA. Lane 4: The supernatant after the third washing of precipitated mRNA (supernatant 3). Lane 5: Purified mRNA. M: DNA ladder, top to bottom, is 10,000, 7000, 4000, 2000, 1000, 500, and 250 (bp). (**c**) RT-qPCR amplification curve and Ct values of three genes (GADPH, S100B, and IL-10) using purified mRNA as the template.

The total RNA was first isolated from cell lysates, and PHA-SAP-oligo(dT) granules were added at a 1:1 ratio to ensure an excess of streptavidin. After the annealing, washing, and elution steps (Figure 5a), the mRNA was purified from total RNA. The concentration and purity of the mRNA were determined by spectrophotometer, as shown in Table 3. The total amount of RNA was 48.45 μg, and 1.26 μg of mRNA was isolated and recovered with an OD_260_/OD_280_ of 1.92, suggesting a relatively high purity. It accounted for 2.59% of total RNA, consistent with the fact that about 1–5% of total RNA is actually mRNA [31]. In addition, other RNAs, like rRNA, the dominant one in the total RNA, were removed and maintained in our supernatants (Table 3).

The result of purified mRNA was visualized by electrophoresis, as shown in Figure 5b. The total RNA showed three typical clear rRNA bands (5 s, 18 s, and 23 s rRNA) with some other fuzzy RNAs (Lane 1), revealing the integrality of RNA. After mRNA was annealed and precipitated with PHA-SAP granules, the supernatant collections contained a large amount of rRNA (Lane 2 and Lane 3, supernatant 0 and 1), while few RNAs (about 0.07 μg of RNA in Table 3) were observed after three times wash (Lane 4, supernatant 3), indicating the washing procedure was effective to achieve a high purity of mRNA. The purified mRNA exhibited a typical band with a comet tail shape between 0.5 and 8 kb, consistent with the previous study [32] (Lane 5). RT-qPCR was successfully performed using the purified mRNA as a template. The Ct values of the inner standard GADPH gene and specifically expressed S100B gene in C6 cells [27] were 21.76 and 20.55, respectively (Figure 5c), while no amplification signal and Ct value for the negative control IL-10 gene. All the results demonstrated the purity and integrality of purified mRNA by PHA-SAP nano-granules, indicating few rRNA or DNA contaminations, less degradation, and good integrity, which were suitable for subsequent experiments.

**Figure 6:**
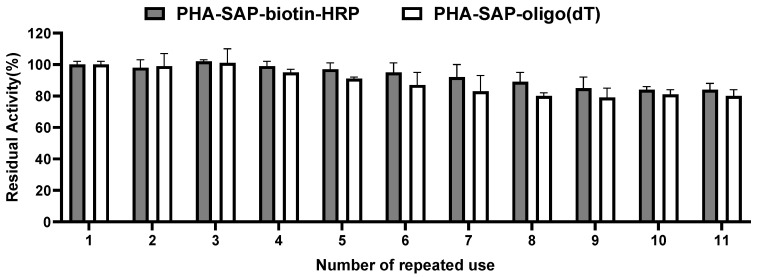
Reusability of PHA-SAP nano-granules. After one cycle of TMB chromogenic reaction (for PHA-SAP-biotin-HRP granules) or mRNA recovery (for PHA-SAP-oligo(dT) granules), the PHA-SAP nano-granules were recycled by centrifugation and subjected to the same procedure and repeated for ten rounds. The initial activity was set to 100%. Error bars indicate SD values from triplicate trials.

The reusability of PHA-SAP nano-granules was also tested by repeated use for ten cycles. As shown in Figure 6, the PHA-SAP nano-granules with biotin-HRP showed relatively stable values after treatment with TMB and retained more than 84% of their initial value after ten repeated uses. It is the same case for PHA-SAP-oligo(dT) nano-granules for mRNA recovery. PHA-SAP-oligo(dT) nano-granules exhibited stable recovery efficiency of mRNA, achieving more than 80% of its initial purified mRNA amount after repeated use (1.01 μg compared to the initial 1.26 μg). The good reusability of PHA-SAP nano-granules will be beneficial for the various and cost-effective bioseparation processes.

### 3.5. Preparation of the Magnetic PHA Nano-Granules for the Fast Recovery

The glycerol gradient-based ultracentrifugation was commonly employed for the purification of PHA nano-granules, which was time-consuming and had high equipment dependency. Our streptavidin-decorated PHA-SAP nano-granules can be further modified with biotinylated magnetic beads (Fe_3_O_4_) on the surface to realize the magnetic recovery and repeated use of PHA nano-granules.

The producer of PHA-SAP nano-granules was cultured and disrupted, and the cell lysates were incubated with biotinylated Fe_3_O_4_ for 1 h, which was further treated by an external magnetic separator. The turbidity of the supernatant was significantly reduced, indicating that the magnetic PHA granules in the cell lysates were collected after magnetic treatment. The magnetic separation of PHA granules from cell lysate was also quantitated by GC analysis. As shown in Table 4, the total amount of PHA in 50 mL of cell lysate was about 61.49 mg, while the PHA content in magnetic PHA granules was about 28.12 mg, accounting for 45.73%, with the remaining ones in the supernatant collections, about 32.55 mg. The supernatant generated by the repeated washing steps was to remove the unspecific absorptions of PHA nano-granules with magnetic beads. A loss of ~1.33% unavoidably occurred in the magnetic recovery procedure. The results confirmed that the PHA nano-granules can be directly recovered from the cell lysates by magnetic treatment, although the recovery efficiency needs improvement.

**Figure 7:**
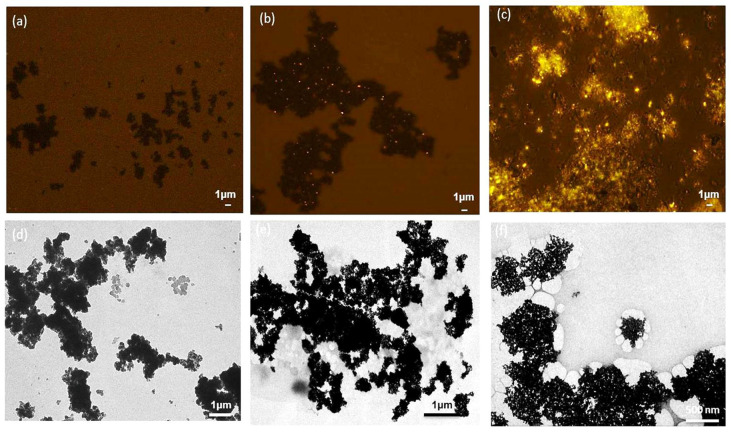
The structures of the magnetic PHA-SAP nano-granules observed by fluorescence microscopy after Nile blue staining and TEM observation. (**a**,**d**): Fluorescence microscopy (**a**) and TEM observation (**d**) of the blank magnetic beads. (**b**,**e**): Fluorescence microscopy (**b**) and TEM observation. (**e**) of the complex of the magnetic beads and the PHA blank granules without streptavidin on the surface. (**c**,**f**): Fluorescence microscopy (**c**) and TEM observation (**f**) of the magnetic PHA-SAP nano-granules.

Fluorescence microscopy after Nile Blue staining and TEM observation also confirmed the structure of the magnetic PHA nano-granules. After Nile Blue staining, the black magnetic beads and bright yellow PHA granules were observed to form a “honeycomb” structure under fluorescence microscopy, and the PHA content was relatively high (Figure 7c). The TEM results also showed the magnetic beads were surrounded by white PHA nano-granules, forming a tight complex (Figure 7f), which was not the same case for the blank magnetic beads in Figure 7a,d. The tight formation of magnetic PHA nano-granules enabled their fast recovery and purification by the magnetic field. Meanwhile, Figure 7b,e showed a few unspecific adsorptions between the magnetic beads and the PHA blank granules without streptavidin on the surface, which is not favorable and can be reduced by repeated washing procedures, or suitable preparation methods and coating for improved stability and dispersibility [33].

## 4. Discussion

Advances in biotechnology and bio-manufacturing have driven a recent reassessment of bioseparation strategies. With the growing cost of downstream purification procedures, it is necessary to develop simple and low-cost bioseparation strategies. Among them, magnetic separations, usually based on nano-beads, favorably gain attention for their fast, gentle, and easy process, and numerous techniques have been developed for this purpose.

The interaction between streptavidin and biotin represents one of nature’s strongest non-covalent bindings, and the streptavidin–biotin system has been extensively used in bioseparation, quantification, and localization of biomolecules in vivo or in vitro [6]. However, the immobilization of streptavidin on chromatography or beads for bioseparation purposes is usually time-consuming and, more importantly, easy to cause the inactivation of streptavidin. In our study, an in situ and one-step immobilization of streptavidin on the intracellular PHA nano-granules has been developed. These nano-granules are produced as fully functional, insoluble polyester inclusions that can be easily separated from the cell. This simple production of streptavidin-decorated nano-granules provides a tantalizing alternative to current commercial functional beads, for which proteins must be expressed, purified, and then chemically attached to solid supports [8].

Different from the previous attempt that streptavidin was displayed on the surface of PHA via fusion with PhaC, another protein that attached to the PHA surface, PhaP, was selected as the adaptor for streptavidin immobilization on PHA in our study. PhaP is an amphipathic protein that adsorbed on the PHA surface via strong hydrophobic interaction, which regulates the number and size of granules and occupies about 5% of physical conditions [13,34]. Compared to PhaC, PhaP is in higher abundance and smaller in size and is not responsible for the PHA accumulation as well, which will avoid the possible inhibition of PHA synthesis. Moreover, compared to the PhaC dimer, the monomeric and simple structure of PhaP will benefit the correct folding of streptavidin when immobilized on the PHA surface. An engineered monomeric streptavidin was previously reported to maintain a high affinity with biotin [18,35], which is considered the ideal variant for the in vivo production of streptavidin-decorated PHA nano-granules beads in our study because the complicated homotetramer structure of wild-type streptavidin is easy to form inactivated aggregates [6].

The plasmid pET28a-SAP containing the monomeric streptavidin and *phaP* fusion gene was successfully constructed and co-transformed into *E. coli* BL21(DE3) with PHA synthetic plasmid pCDF-ABC (Figure 2). The producer demonstrated efficient expression of the streptavidin-PhaP fusion protein by SDS-PAGE analysis of whole cell lysates. The recombinant *E. coli* harboring two expression plasmids produced 1.95 g/L of PHA nano-granules after 48 h cultivation in MMG medium, which was confirmed by Nile blue A staining observation and TEM observation (Figure 3). The yield was a little higher than that of streptavidin-decorated PHA granules achieved by streptavidin fusing with PhaC [8], demonstrating that fusion with PhaP favored an increased accumulation of PHA nano-granules.

The PHA nano-granules were purified by glycerol-gradient-based ultracentrifugation, and the streptavidin-PhaP fusion protein was observed to be dominantly immobilized on the surface of PHA granules, as shown in Figure 4a. There are some other PHA-associated proteins like PhaC and possible contaminated ones on the surface, which showed no inhibitory effect on the functionality of streptavidin, as demonstrated in our further characterization. The biotin-binding affinity of streptavidin on PHA nano-granules was qualitatively and quantitatively characterized by western blot and chromogenic reaction with TMB. The average biotin affinity of PHA-SAP nano-granules was 0.1 ng of biotin/μg surface protein (50 ng of biotin-HRP/mg PHA granules), suggesting that streptavidin on PHA-SAP granules occupied about 6.78% of the total weight of PHA granules. It is a considerable level of surface density and biotin affinity compared to the previous studies using PhaC as an adapter, which showed a surface density of 0.06% to 6.85% of total PHA [19,21]. The result suggested that the abundance of PhaP benefited a high density of immobilized protein on the PHA surface, leading to the full functionality and high biotin affinity of the streptavidin-decorated PHA-SAP nano-granules. As a proof of concept, biotinylated oligo(dT) or magnetic beads were further functionalized to PHA-SAP nano-granules for mRNA isolation or magnetic recovery.

The isolation and purification of mRNA are essential for the blot hybridization and the construction of the cDNA library etc. mRNA is commonly isolated by oligo(dT) cellulose affinity chromatography or oligo(dT) attached magnetic beads via the interaction of oligo(dT) and poly(A) tail of mRNA [31]. The immobilization of oligo(dT) usually requires tedious chemical cross-linking on these solid supports. In our study, the oligo(dT) probe was simply decorated on the PHA nano-granules by the strong interaction of streptavidin and biotin, generating a novel biobased PHA-SAP-oligo(dT) beads for mRNA isolation. About 1.26 μg of mRNA (occupied 2.59%) was purified from 48.45 μg of total RNA in C6 cells. The purified mRNA appeared as a smear between 0.5 and 8 kb with a typical comet tail shape in electrophoresis, consistent with a previous study [32]. The purified mRNA was suitable for subsequent RT-qPCR, which confirmed its good integrity. The other RNA contaminants were removed by repeated washing, achieving the high purity of mRNA, as shown in Table 3 and Figure 5b. The purified mRNA by PHA-SAP-oligo(dT) nano-granules demonstrated a considerable concentration, a high purity with few DNA and rRNA contaminations, and good integrity. Although the PHA-SAP nano-granules were demonstrated to work for total RNA preparations in our study, they can also be used for cell lysates that would otherwise clog standard oligo(dT) cellulose column systems. Our simple and fast platform provides an alternative for efficient and less degradable mRNA isolation.

The streptavidin-decorated PHA-SAP nano-granules were further modified with biotinylated magnetic beads (Fe_3_O_4_) for their fast magnetic recovery directly from cell lysates to avoid the cumbersome and complicated procedures of ultracentrifugation. The cell lysates or PHA nano-granules were well dispersed in solution, incubated with biotinylated Fe_3_O_4_, and then retrieved using an external magnet. The GC analysis, fluorescence microscopy, and TEM observation of the magnetic PHA beads demonstrated the direct recovery of PHA nano-granules from the cell lysates by magnetic treatment, with a recovery efficiency of about 45.73%. Unspecific adsorptions between the PHA blank granules without streptavidin and the magnetic beads were observed in our study, which was significantly reduced by repeated washing steps (Table 4 and Figure 7b,e). Based on the magnetic PHA nano-granules, the procedure of separation and purification of PHA granules was effectively simplified. However, the recovery efficiency is far from satisfactory, mainly due to the poor dispersion of magnetic beads, which exhibited serious aggregation even after ultrasonic homogenization. As a result, the interaction efficiency of PHA-SAP nano-granules and magnetic beads was significantly reduced, leading to the low yield of functional magnetic PHA beads and low recovery efficiency.

Fe_3_O_4_ nanoparticles are widely used in the bioseparation process with superparamagnetism, high susceptibility, and unique biocompatibility. The preparation of well-dispersed biotinylated Fe_3_O_4_ nanoparticles in our study can be efficiently addressed via two major strategies. One attempt is to initially prepare Fe_3_O_4_ nanoparticles using some advanced methods that can generate nanoparticles with higher dispersibility. Various methods have been intensively studied to prepare Fe_3_O_4_ nanoparticles, including co-precipitation, hydrothermal, pyrolysis, sol-gel, microemulsion, sonochemical, electrodeposition, polyol method, etc. [33]. Fe_3_O_4_ nanoparticles in our study were prepared by common co-precipitation with a hydrophobic surface, so they will interact with each other to form larger clusters, eventually resulting in their serious aggregation. Other preferred methods like microemulsion, sol-gel method, and polyol technology can be developed in the initial preparation of Fe_3_O_4_ nanoparticles, and aggregation will be effectively controlled to generate uniform and well-dispersed nanoparticles [33].

Another important attempt is to functionalize the nanoparticles with some coatings to reduce the interaction between magnet beads [4,36]. The surface functionalization of Fe_3_O_4_ nanoparticles is an essential step to improve their stability and reduce unfavorable aggregation, which can be achieved by the modification of organic materials and inorganic materials. Among them, surfactant-functionalized magnetic nanoparticles are commonly employed, as a suitable surfactant helps to form stable and sufficient repulsive interactions in the system. The carboxamide-functionalized superparamagnetic iron oxide nanoparticles (SPIONs) have been prepared with high water solubility and water stability due to the hydrophilic carboxamide group [37]. It is showed the cetyltrimethylammonium bromide (CTAB) and benzyl-secondary ammonium chloride (BKC) modification of Fe_3_O_4_ nanoparticles can effectively reduce the particle size and enhance their stability in solution [38]. A polydimethylsiloxane (PDMS) and poly(polyethylene glycol) methacrylate (PPEGMA) amphiphilic bilayer surfactants for surface modification was reported, and the hydrophilic PEG not only provides space repellency to particles but also improves water dispersibility. Additionally, other organic materials such as polymers PEI and PAA for surface functionalization provide an electrostatic repulsion effect for enhanced polydispersity of magnetic nanoparticles [39], and some small hydrophilic molecules like oleic acid, citric acid, or L-cysteine [40], as well as inorganic materials like silicon dioxide (SiO_2_), can also contribute the stability of Fe_3_O_4_ nanoparticles [41]. All in all, the above protective functionalized coatings, which will usually form a core-shell micro-structure, will significantly improve the stability and dispersibility of Fe_3_O_4_ nanoparticles, which will eventually benefit the fast magnetic recovery of PHA-SAP nanogranules in our study.

Some commercial streptavidin magnetic particles have been commercialized by companies such as Dynal (Dynalbeads M-270, Dynal Biotech, Oslo, Norway), Miltenyi, and GoldMag [36], in which streptavidin is traditionally assembled by either physical adsorption or chemical linking on the surface of magnetic beads. Instead, magnetic beads were displayed around the biopolymer granules in our magnetic PHA-SAP nano-granules, forming a novel “core-shell” structure of magnetic nano-granules, which has also been achieved in superparamagnetic nanobeads using a ferrous fluid and applied in the early sensitive diagnostics of cancer [15]. Compared to other magnetic particles, our PHA-SAP nano-granules are more scalable as they can be manufactured in a range of recombinant biological hosts and are able to offer better production yields over biomass. Additionally, the one-step functionalization and production of PHA-SAP nano-granules in vivo minimize the activity loss of streptavidin and exhibited structurally stability and a high efficiency [42].

Our study provides an expandable nano-granules platform for various bioseparation purposes. The magnetic PHA-SAP nano-granules can be further functionalized with other biotinylated biomolecules, such as antibodies or ligands, to serve as bioseparation resin for various molecules or chemicals. A similar study has been reported to functionalize PHA nano-granules with biomarker-specific antibodies for the isolation and detection of both methylated DNA and exosome cancer biomarkers/exosome. The magnetic PHA nano-granules exhibited cost-effective manufacturing, remarkable stability, and high analytical performance in the detection of methylated DNA for rapid epigenetic evaluations in human plasma matrices and cancer cell lines, as well as served for fast detection and isolation of exosomes with a sample volume as low as 5 μL and an electrochemical signal within 2 h [15]. Additionally, functional PHA beads were also successfully applied for the stable purification of biotinylated DNA fragments with different lengths, as well as to bind biotinylated antibodies (goat polyclonal to rabbit IgG H&L antibody) for ELISA detection and flow cytometry analysis with a strong increase in fluorescence intensity (78%) compared to the control [8]. Moreover, the immunoglobulin G (IgG) was also anchored on the surface of PHA nano-granules, demonstrating improved IgG-binding capacity and purification power of biomolecules to commercial protein A sepharose [43]. Although the strategies for PHA functionalization may vary in the above cases, they reveal the expandability and versatility of the PHA nano-granules platform for various purification tasks in complicated systems. With an increase in artificial intelligence (AI) generated molecules and chemicals, our PHA nano-granules can also be further decorated with biotinylated specific ligands for the screening and fishing of a huge number of AI-generated candidates, such as binding of receptor binding domain (RBD) of the spike protein of COVID-19 as a bait for the possible small molecule or neutralizing antibody for COVID-19 treatment. Besides applications in bioseparation, PHA nano-granules can be functionalized with a variety of biomolecules to serve as powerful tools for multiple purposes, such as protein production and purifications, vaccines, diagnostic imaging, and enzymatic catalysis for plenty of bioproducts like D-allulose, 3,4-dihydroxyphenyl-L-alanine (L-DOPA) etc., as reported in our previous study [10,19,44].

The magnetic PHA nano-granules in our study can serve as bioseparation resin for large-scale purification processes. However, there are still challenges to be addressed for their industrial-scale production and applications.

Simple purification strategies of PHA nano-granules itself: The common purification method for PHA nano-granules is glycerol-gradient-based ultracentrifugation (100,000× *g* for 2.5 h), which is tedious and time-consuming, and it can barely be achieved in industrial production. Breakthrough is urgent to develop a simple purification procedure that is suitable for large-scale. Magnetic recovery in our and other reported studies is beneficial for the rapid purification of PHA nano-granules. Additionally, separation or catalysis directly by cell lysates containing our nano-granules has been demonstrated feasible, especially when an ultra-high content of PHA granules (up to 80–90 wt%) was produced in the *Halomonas bluephagenesis* host [19]. More strategies are under intensive study to avoid complicated purification processes of these promising nano-granules, which also need multidisciplinary knowledge.

Enhanced specificity towards target molecules: Industrial bioseparation is challenging due to non-specific adsorption or contamination risks in complex biological samples, which could affect purity and specificity during bioseparation. The interaction between streptavidin and biotin, one of nature’s strongest non-covalent bindings, was used in our PHA nano-granules platform, while the high affinity of biotinylated molecules to targets in the complicated samples is the focus to reduce non-specific binding. Roughly, several methods have been developed for specific ligand screening of target molecules, including Surface Plasmon Resonance (SPR), Bio-Layer Interferometry (BLI), Microscale Thermophoresis (MST) system, and Systematic Evolution of Ligands by Exponential enrichment (SELEX) etc., offering flexible, powerful, and rapid methods for measuring and high-throughput screening of highly specific interactive molecules [45,46].

Reduced cost of the whole process of production and bioseparation: A cost-effective process of PHA nano-granules based bioseparation relies on two steps: the low-cost production of PHA nano-granules and an economic bioseparation procedure. Recently, there have been several breakthroughs for cost reduction for PHA production, including the low-cost halophilic producer *Halomonas bluephagenesis* and its corresponding “Next-Generate Industrial Biotechnology (NGIB)”, which will remarkably reduce the PHA production cost by up to about 30% (2.9 USD/kg for NGIB vs. 4.0 USD/kg for traditional biotechnology) [11]. Meanwhile, our previous study demonstrated that *H. bluephagenesis* is a great host for the production of PHA nano-granules with its increased cell growth, PHA accumulation, and remarkably improved content and activity of functionalized proteins, beneficial for the next bioseparation. Moreover, the cost of the bioseparation procedure can be reduced by the simple purification strategies mentioned above and the good repeatability of our nano-granules, as demonstrated in Figure 6.

Artificial intelligence integrates interdisciplinary knowledge: The production and applications of functional PHA-SAP nano-granules require interdisciplinary knowledge from microbiology, molecular biology, chemical engineering, chemistry, and material science. Therefore, bridging interdisciplinary boundaries between researchers from different fields should be encouraged. Moreover, the integration of AI in the design, control, and optimization of PHA nano-granules will significantly accelerate their applications.

Biosafety: Currently, most PHA nano-granules, including our study, were produced in *E. coli*, which may face potential biosafety challenges, especially problematic for medical applications due to the potential for endotoxin contamination. The alternatives are the generally-regarded-as-safe bacterium *Lactococcus lactis* and toxin-free *H. bluephagenesis*, which have demonstrated success in PHA nano-granules based vaccines and L-DOPA production [10]. After purification from the microbial host, the PHA nanomaterial itself offers advantages such as biodegradability of the non-toxic natural scaffold and great biocompatibility, providing sustainable and environmentally friendly bioprocessing for humans and the environment.

In conclusion, a novel strategy of bioseparation based on the streptavidin-decorated PHA nano-granules was developed in this study. The in situ and one-step immobilization of streptavidin on PHA granules was achieved by the fusion of streptavidin to the N-terminus of PhaP, and the resulting PHA-SAP nano-granules demonstrated a high streptavidin density on the PHA surface and a considerable affinity to biotin. The PHA-SAP nano-granules were further functionalized with biotinylated oligo(dT) and biotinylated magnetic beads as a proof of concept, showing their good functionalities in mRNA isolation and rapid magnetic recovery. The advantages of a simple preparation process, high density and activity of streptavidin, stable and strong interaction to target biotinylated molecules, and good expandability, making the novel PHA-SAP nano-granules an efficient platform for various bioseparation purposes. Much of their potential remains untapped, especially in achieving the successful “bench-to-factory” translation that still requires rigorous optimization to meet industry standards, which will be addressed in the future.

## Figures and Tables

**Table 1:** Strains, plasmids, and oligonucleotides in this study.

Name	Descriptions	Source
Strains		
*Escherichia coli* DH5α	*F-φ-5dlacZΔlac Δ*(*lacZYA-argF*)*U169 deoR recA1 endA1 hsdR17*(*Rk*+, *mK*+) *phoA supE44 λ-thi-1 gyr96 relA1*	Takara, Beijing, China
*Escherichia coli* BL21(DE3)	*F-ompT hsdSB*(*rB-*, *mB-*)*gal dcm* (*DE3*)	Novagen, Madison, WI, USA
Plasmids		
pET28a	Kan^r^, efficient expression vector with T7 promoter	Invitrogen, Carlsbad, CA, USA
pET28a-SAP	Kan^r^, expression vector for streptavidin-PhaP fusion protein under T7 promoter	This study
pCDF-ABC	Sm^r^, plasmid for the production of PHA nano-granules, containing the PHA synthetic gene cluster *phaABC* from *Ralstonia eutropha* under the control of T7 promoter	[17]
Primers		
SA-F	CATGCCATGGGCATGACCACCGTGAGCATTACC	This study
SA-R	AGCTCTTGATCACGTCCATATTGCTACCACCTGAACCACCAC	This study
PhaP-F	GTGGTGGTTCAGGTGGTAGCAATATGGACGTGATCAAGAGCT	This study
PhaP-R	ATCGCTCGAGTCAGGCCTTGCCCGTGCTCT	This study

**Table 2:** MMG medium formulation.

Type	Components	Concentration (g/L)
Substrate	glucose	20
	yeast extract	1
Component I (50×)	Na_2_HPO_4_·H_2_O	482.5
	KH_2_PO_4_	75
Component II (50×)	(NH_4_)_2_SO_4_	100
	MgSO_4_·7H_2_O	20
Component III (100×) Prepare with 1 mol/L HCl	Fe(III)-NH_4_-Citrate	5
	CaCl_2_·2H_2_O	2
	ZnSO_4_·7H_2_O	0.1
	MnCl_2_·4H_2_O	0.03
	H_3_BO_3_	0.3
Component IV (1000×) Prepare with 1 mol/L HCl	CoCl_2_·6H_2_O	0.2
	CuSO_4_·5H_2_O	0.01
	NiCl_2_·6H_2_O	0.02
	NaMoO_4_·2H_2_O	0.03

**Table 3:** Efficiency of PHA-SAP nano-granules for mRNA isolation.

	Concentration (ng/μL)	OD_260_/OD_280_	Amount of RNA (μg)	Percentage of the Total RNA
Total RNA	1938.07 ± 19.42	1.89 ± 0.06	48.45 ± 0.49	100%
Supernatant 0	1241.60 ± 11.57	1.88 ± 0.02	31.04 ± 0.29	64.06%
Supernatant 1	109.43 ± 6.01	1.90 ± 0.07	2.74 ± 0.15	5.65%
Supernatant 3	2.70 ± 0.47	1.95 ± 0.01	0.07 ± 0.01	0.14%
Purified mRNA	50.25 ± 6.29	1.92 ± 0.05	1.26 ± 0.16	2.59%

**Table 4:** Efficiency of magnetic recovery of PHA nano-granules.

	Amount of PHA by GC Analysis (mg)	Percentage
50 mL cell lysates	61.49 ± 0.91	100%
Magnetic PHA nano-granules	28.12 ± 2.23	45.73%
Supernatant collections	32.55 ± 1.41	52.93%

## Data Availability

The original contributions presented in this study are included in the article. Further inquiries can be directed to the corresponding authors.
